# Multi-Omics Approaches Against Abiotic and Biotic Stress—A Review

**DOI:** 10.3390/plants14060865

**Published:** 2025-03-10

**Authors:** Venkatramanan Varadharajan, Radhika Rajendran, Pandiyan Muthuramalingam, Ashish Runthala, Venkatesh Madhesh, Gowtham Swaminathan, Pooja Murugan, Harini Srinivasan, Yeonju Park, Hyunsuk Shin, Manikandan Ramesh

**Affiliations:** 1 Department of Biotechnology, PSG College of Technology, Coimbatore 641004, India; 21b256@psgtech.ac.in (V.M.);; 2 Indian Council of Agricultural Research (ICAR), National Institute for Plant Biotechnology (NIPB), PUSA Campus, New Delhi 110012, India; vinoth.radhika07@gmail.com; 3 Division of Horticultural Science, College of Agriculture and Life Sciences, Gyeongsang National University, Jinju 52725, Republic of Korea; shinpomo@gnu.ac.kr; 4 Department of GreenBio Science, Gyeongsang National University, Jinju 52725, Republic of Korea; 5 Department of Basic Sciences, School of Science and Humanities, SR University, Warangal 506371, India; ashish.runthala@gmail.com; 6 Department of Biotechnology, Alagappa University, Karaikudi 630003, India; mrbiotech.alu@gmail.com

**Keywords:** abiotic stress, biotic stress, crop plants, CRISPR-Cas9, multi-omics

## Abstract

Plants face an array of environmental stresses, including both abiotic and biotic stresses. These stresses significantly impact plant lifespan and reduce agricultural crop productivity. Abiotic stresses, such as ultraviolet (UV) radiation, high and low temperatures, salinity, drought, floods, heavy metal toxicity, etc., contribute to widespread crop losses globally. On the other hand, biotic stresses, such as those caused by insects, fungi, and weeds, further exacerbate these challenges. These stressors can hinder plant systems at various levels, including molecular, cellular, and development processes. To overcome these challenges, multi-omics computational approaches offer a significant tool for characterizing the plant’s biomolecular pool, which is crucial for maintaining homeostasis and signaling response to environmental changes. Integrating multiple layers of omics data, such as proteomics, metabolomics, ionomics, interactomics, and phenomics, simplifies the study of plant resistance mechanisms. This comprehensive approach enables the development of regulatory networks and pathway maps, identifying potential targets for improving resistance through genetic engineering or breeding strategies. This review highlights the valuable insights from integrating multi-omics approaches to unravel plant stress responses to both biotic and abiotic factors. By decoding gene regulation and transcriptional networks, these techniques reveal critical mechanisms underlying stress tolerance. Furthermore, the role of secondary metabolites in bio-based products in enhancing plant stress mitigation is discussed. Genome editing tools offer promising strategies for improving plant resilience, as evidenced by successful case studies combating various stressors. On the whole, this review extensively discusses an advanced multi-omics approach that aids in understanding the molecular basis of resistance and developing novel strategies to improve crops’ or organisms’ resilience to abiotic and biotic stresses.

## 1. Introduction

The integration of omics approaches, encompassing transcriptomics, genomics, proteomics, metabolomics, and phenomics, represents a transformative strategy for understanding plant resilience against biotic and abiotic stresses. This multi-omics framework allows researchers to investigate the composite interactions between various biological molecules and their roles in stress responses, leading to more effective crop improvement strategies [1]. Genomics focuses on the complete set of DNAs within an organism. In the context of abiotic and biotic stress, genomics helps identify quantitative trait loci (QTLs) that confer resistance to specific stresses. Advanced techniques such as genome-wide association studies (GWASs) enable the identification of genetic variations linked to desirable traits, facilitating marker-assisted breeding programs to develop resilient crop varieties [2].

Transcriptomics analyzes the composite set of RNA transcripts produced by the genome under specific conditions. Researchers can identify key regulatory genes involved in stress tolerance by studying gene expression patterns during stress conditions. This knowledge is crucial for understanding how plants adapt at a molecular level and identifying potential genetic targets for crop improvement [3]. Proteomics examines the entire complement of proteins expressed in a plant. This approach provides insights into how proteins function and interact during stress responses. Techniques such as mass spectrometry allow for identifying and quantifying proteins involved in signaling pathways that activate defense mechanisms against environmental stresses. Metabolomics refers to the comprehensive analysis of metabolites within a biological sample. Phenomics focuses on the observable traits resulting from the interaction between an organism’s genotype and environment. High-throughput phenotyping technologies enable real-time monitoring of plant responses to abiotic stresses such as drought or salinity, allowing researchers to correlate physiological traits with molecular data [4].

Recent advancements have emphasized the importance of integrating data from multiple omics layers to gain a holistic comprehension of plant stress responses. By employing systems biology frameworks, researchers can model complex interactions among genes, proteins, and metabolites during stress responses. Unsupervised and semi-supervised ML algorithms effectively analyze plant traits shaped by genotype–environment interactions without needing large labeled datasets. At the same time, decision tree ensembles excel in genomic prediction and multi-omics integration [5]. Advances in high-throughput sequencing and analysis techniques have accelerated data generation across various omics platforms. This rapid data acquisition gives timely insights into how plants adapt to changing environmental conditions [2]. Integrating omics data with functional genomics approaches aids in elucidating gene functions related to stress tolerance. For example, recent studies have identified transcription factors (TFs) that regulate gene expression under abiotic stress conditions, providing targets for genetic manipulation [3].

Multi-omics data can inform breeding programs by identifying resilient genotypes that exhibit desirable traits under stress conditions [1]. This information can be used to develop climate-smart crops that maintain yield stability despite environmental challenges. Understanding the molecular mechanisms underlying stress tolerance enables targeted biotechnological interventions, such as genetic engineering or CRISPR-based modifications aimed at enhancing specific traits related to abiotic or biotic resistance [6,7]. By developing crops that can withstand adverse conditions such as drought or salinity, integrated omics approaches contribute to sustainable agricultural practices that ensure food security in a changing climate [8,9].

**Figure 1:**
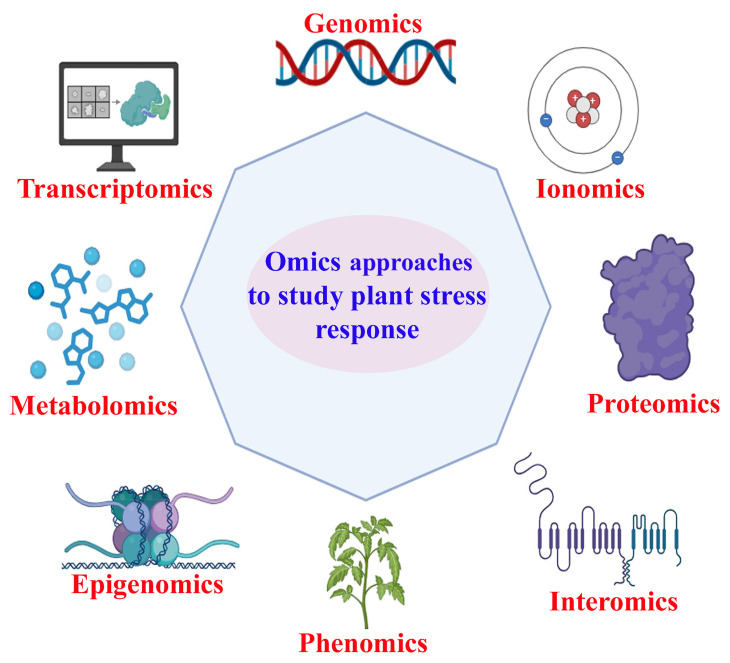
Omics approaches to studying plant stress response.

The integration of omics (Figure 1) approaches represents a powerful strategy for enhancing plant resilience against abiotic and biotic stresses. Researchers can develop a comprehensive understanding of plant resilience mechanisms by leveraging insights from genomics, transcriptomics, proteomics, metabolomics, and phenomics [1]. Recent advancements in technology and data analysis further enhance this integration, paving the way for innovative solutions that improve crop performance under challenging environmental conditions. As these approaches continue to evolve, they promise to develop resilient crops capable of thriving in an increasingly unpredictable climate [3,7].

## 2. Proteomics Against Abiotic and Biotic Stresses

Proteomics is an important method for studying plant stress tolerance because it provides information about protein expression changes and the pathways involved in abiotic stress responses [8]. This high-throughput approach enables the profiling of the entire proteome and aids in understanding plant functional, structural, and developmental dynamics under various environmental conditions [10,11]. Comparative proteomics, a prominent subfield, allows protein profiling across individuals exposed to stress treatments, revealing proteins and pathways critical for adaptation [12]. Proteins are central to stress response mechanisms, acting as key regulators of cellular homeostasis and physiological processes that drive the formation of new stress-adaptive phenotypes [13].

Rice development and growth are greatly impacted by salt stress, which makes a better understanding of tolerance mechanisms necessary to increase crop resilience. Proteomic analysis reveals important proteins and their functional annotations, providing vital information on the genetic variety driving salt tolerance. During exposure to salt stress, temporal proteome investigations aid in the clarification of precisely calibrated stress-responsive pathways. Combining multi-omics techniques like transcriptomics, metabolomics, and genomes with high-throughput proteomics offers a complex understanding of the molecular networks with salt stress adaptation [12,13]. Significant proteome changes in salt-stressed rice seedlings have been brought to light by recent developments in rice proteomics, which have also identified potential target enzymes, proteins, and genes for breeding salt-tolerant genotypes and adaptive techniques. However, problems like data integration, resource-intensive analysis, and candidate functional validation still exist [13].

Proteomics, which can be classified into structural, sequence, and phosphoproteomics, is the study of all expressed proteins in an organism [14]. Structural proteomics aims to determine the functions of proteins by examining their structures using methods like electron microscopy, X-ray diffraction, nuclear magnetic resonance (NMR), crystallization, and computer-based modeling [15]. Protein microarrays and yeast hybrid systems are two techniques used in functional proteomics to study the functions of proteins [16]. Early proteomic changes associated with individual abiotic stress remain to be elucidated, which will allow the identification of distinct sets of effectors for stress signaling. The function of these early effectors may be masked by secondary processes at a later stage [17]. By identifying phosphorylated proteins and their amino acid residues, phosphoproteomics analyzes protein phosphorylation and aids in the understanding of how crops react to stress [18]. The discovery of stress-resistant agricultural varieties has been made possible by combining this method with proteomics to investigate crops such as wheat and grapevine under disease and drought stress [10]. The protein composition of rice kernels has been crucially elucidated by detailed research employing cutting-edge proteomic techniques, such as gel-free shotgun methods and two-dimensional polyacrylamide gel electrophoresis (2D-PAGE) [19]. Most proteomic studies have utilized gel-based separation approaches, which primarily identify highly abundant proteins. The use of advanced LC-based separation techniques may significantly improve the detectability of low-abundance proteins such as TFs, kinases, and transport proteins [17].

Recent developments in proteomic techniques have greatly improved the knowledge of the molecular processes that enable rice’s ability to tolerate heat [20]. Methods such as isobaric tags for relative and absolute quantification (iTRAQ) and tandem mass tag (TMT) labeling now allow for precise measurement and identification of heat-responsive proteins. Heat shock proteins (HSPs), essential proteins that preserve cellular homeostasis under stress, have been discovered using TMT labeling and high-resolution mass [21]. Similar to TMT, studies using iTRAQ have revealed new pathways that help heat-tolerant rice types become more resilient, such as signal transduction, protein folding, and oxidative stress response [22]. To enhance heat tolerance, research on post-translational modifications (PTMs) such as phosphorylation, ubiquitination, and acetylation has shown how such modifications modulate protein function and stability under stress [23]. By identifying essential proteins and connections for possible genetic engineering and breeding programs, advances in bioinformatics, systems biology, and computational biology—including machine learning and network analysis—have advanced our understanding of the regulatory networks of heat stress responses. Combined, these innovative methods present feasible approaches for creating rice cultivars that tolerate heat by precisely modifying stress-responsive proteins and pathways [20].

Mass spectrometry (MS) technology advancements have greatly improved the ability to detect and measure proteins in diverse mixtures. Shotgun proteomics, sometimes called the bottom-up strategy, is one method that involves breaking down proteins into peptides by enzymes and then analyzing them using mass spectrometry. This method allows for accurate protein quantification, especially with triple-quadrupole mass spectrometry (MS), when paired with instruments such as selective reaction monitoring (SRM) and multiple reaction monitoring (MRM) [24]. Accurate peptide breaking and sequencing are facilitated by molecular mass assessment methods such as collision-induced dissociation (CID) and electrospray ionization (ESI) [25]. Among high-throughput techniques, liquid chromatography–tandem mass spectrometry (LC-MS/MS) and matrix-assisted laser desorption/ionization time-of-flight mass spectrometry (MALDI-TOF MS) are frequently employed because they have high detection capabilities [26] and make de novo protein sequence assembly easier. Advances such as Fourier transform MS and Orbitrap-based MS further improve resolution and mass accuracy, supporting complex proteomics studies [27]. These advancements, combined with bioinformatics tools, have greatly enhanced the understanding of proteome dynamics and biological systems [26,27].

While an integrated approach combining proteomics, genomics, and other allied omics branches of biotechnology has shown promise in advancing crop improvement programs globally, the effective integration and interpretation of results from vastly different techniques remain a challenge, as current AI and machine learning frameworks are still evolving to master these complexities [28]. The fast-growing advancements in omics databases are expected to play a key role in shaping next-generation crops, emphasizing the need for their strategic application to benefit farmers. Omics technologies generate invaluable data that drive numerous biotechnological applications, enhancing our understanding of complex plant systems [28]. Recent breakthroughs in proteomic platforms, alongside related advancements in plant biotechnology, offer innovative strategies that encourage plant scientists to incorporate these approaches into crop improvement programs [29]. These advancements hold immense potential to revolutionize agricultural practices and address global food security challenges.

## 3. Metabolomics Against Abiotic and Biotic Stresses

As the global population is projected to reach 9.7 billion by 2050, the demand for food production is expected to increase by 70% [30]. Various biotic stresses, such as pests and diseases, and abiotic stresses, including climate change-related factors like drought, soil salinity, and extreme weather conditions, compound this challenge. Traditional agricultural practices, heavily reliant on chemical fertilizers and pesticides, have raised significant environmental concerns, prompting a shift towards more sustainable practices [30].

### 3.1. The Role of Metabolomics

Metabolomics, the comprehensive study of metabolites within an organism, is emerging as a vital approach to understanding how plants respond to these stresses. By analyzing the metabolic pathways that are altered under stress conditions, researchers can identify specific metabolites that play crucial roles in plant defense mechanisms [31]. This understanding can help in creating innovative strategies to improve plant resilience.

Metabolomics represents a high-throughput and comprehensive evaluation of metabolites in a plant system, offering insights into stress responses that cannot be captured by genomics or transcriptomics alone. Techniques like GC-MS and LC-MS are invaluable for detecting a broad range of metabolites, including primary metabolites (sugars, amino acids) and secondary metabolites (flavonoids, alkaloids) that play crucial roles in plant defense [32].

### 3.2. Metabolomics in Biotic Stress

Biotic stress in plants arises from living organisms such as pathogens, pests, and herbivores. These stressors lead to significant plant metabolic reprogramming, activating defense mechanisms that help mitigate damage and enhance survival. Metabolomics provides a powerful approach to understanding these biochemical responses, offering insights into the plant’s adaptive strategies to cope with such challenges [30].

Research has shown that plants such as *Arabidopsis thaliana* and *Solanum lycopersicum* (tomato) can swiftly activate their defense pathways when pathogens invade, leading to the rapid production of phytoalexins and other antimicrobial compounds [33]. For example, studies have documented increased phenolic compounds following fungal infections, which are crucial for reinforcing plant defenses. Phenolics are well known for their role in enhancing structural barriers and exhibiting antimicrobial properties, effectively preventing the spread of pathogens. These compounds are synthesized in response to stress, underscoring an essential aspect of plant immunity and resistance [34].

#### 3.2.1. Defense Mechanisms

Chemical defenses: Plants synthesize various secondary metabolites as part of their defense response to biotic stress. Among these, phenolic compounds are one of the key groups produced when plants are exposed to biotic stressors. These compounds are essential in strengthening physical barriers, such as cell walls, and exhibiting antimicrobial properties that help prevent pathogen entry [31,34]. In *A. thaliana*, for instance, phenolic levels increase after infection by fungal or bacterial pathogens, which helps to boost resistance to pathogens like *Pseudomonas syringae* [35]. Phytoalexins, another class of antimicrobial compounds, are synthesized de novo in response to pathogen attacks [33]. In tomatoes, *Botrytis cinerea* infection induces the production of other phytoalexins, such as rishitin, which helps mitigate the effects of the pathogen and supports plant survival [36].

Insect herbivory: Insect herbivory activates plant defense mechanisms by producing volatile organic compounds (VOCs), which serve as signals to neighboring plants and attract natural predators. For example, when maize (*Zea mays*) is attacked by the fall armyworm (*Spodoptera frugiperda*), it produces elevated levels of VOCs, particularly terpenes [37]. These VOCs help the plant defend itself and serve as signals to nearby plants, alerting them to prepare for a potential attack. Additionally, VOCs attract natural predators of herbivores, creating a defense network that minimizes the damage to the plant [37].

#### 3.2.2. Pathogen-Specific Responses

Plants activate distinct metabolic pathways in response to different pathogens, reflecting the specificity of their defense mechanisms. For example, when challenged by *Fusarium head blight*, a fungal pathogen, resistant barley cultivars exhibit increased production of flavonoids, phenylpropanoids, fatty acids, and terpenoids. These metabolites play a dual role in enhancing resistance: they inhibit pathogen proliferation and reinforce the structural integrity of plant cells [38,39]. Such metabolic reprogramming is a cornerstone of the plant’s defense strategy, highlighting the critical role of metabolomics in deciphering biotic stress responses [40].

Genetic factors further modulate plant resistance through quantitative trait loci (QTLs). For instance, the *Qfhs.ndsu-3BS* locus in wheat is associated with resistance traits, including detoxifying mycotoxins produced by pathogens. These QTLs correlate with unique metabolomic signatures, offering insights into the genetic underpinnings of plant defense [41].

#### 3.2.3. Organ-Specific Metabolomic Changes

Metabolomic responses are highly tissue-specific, reflecting the varying roles and exposure levels of different plant organs. Roots, for example, often accumulate higher concentrations of defense-related metabolites compared to leaves during pathogen attacks, likely due to their direct contact with soil-borne pathogens and their critical role in nutrient uptake. A study on *Quercus* species infected with *Phytophthora cinnamomi* demonstrated this organ-specific response: roots exhibited a significant decline in sugars like D-glucose and D-fructose, while stress-related metabolites such as L-proline increased in both roots and leaves, with a more sustained elevation in roots [42]. This differential metabolic response ensures that vital organs like roots are prioritized for defense, safeguarding overall plant health and survival. Organ-specific metabolomic profiling has also been employed to study Indian mustard (*Brassica juncea*), revealing distinct metabolite distributions across seeds, flowers, and leaves. Specific metabolites were found to be enriched in particular tissues, underscoring the strategic allocation of defense-related compounds [43]. Such insights are invaluable for designing targeted breeding programs aimed at enhancing resistance traits in crops.

### 3.3. Integration of Omics Technologies

- The integration of metabolomics with other omics disciplines, such as transcriptomics and proteomics, provides a multi-dimensional perspective on plant stress responses. Metabolomics captures dynamic changes in metabolite profiles, while transcriptomics reveals shifts in gene expression, and proteomics identifies the proteins involved in defense mechanisms. Together, these approaches unravel the complex regulatory networks that govern plant responses to stress [44].
- For example, integrated omics studies have shown that changes in gene expression do not always correlate directly with metabolic adaptations, indicating the presence of additional regulatory layers [45,46]. This highlights the necessity of combining multiple omics technologies to achieve a comprehensive understanding of plant stress responses, paving the way for more effective strategies to enhance crop resilience.

### 3.4. Variability Among Cultivars

- Genetic diversity among cultivars of the same species results in distinct metabolomic profiles when exposed to biotic stress. For instance, resistant cultivars often produce higher levels of specific metabolites, such as flavonoids and terpenoids, compared to susceptible cultivars [47,48]. These metabolites play a significant role in enhancing resistance to pathogens, highlighting the importance of genetic variability in developing crop varieties capable of withstanding biotic stress. Harnessing this diversity can lead to the improvement of crop resistance, ensuring better yields despite environmental challenges.
- Metabolomics is a critical tool for identifying metabolic traits linked to biotic stress resistance. These insights can support breeding programs for more resistant crop varieties [49]. By selecting specific metabolic traits that confer resistance, breeders can accelerate the creation of crops better suited to face challenges posed by pests, pathogens, and herbivores. In this way, metabolomics facilitates precision breeding, promoting the development of resilient crops for sustainable agriculture [50].

### 3.5. Abiotic Stress and Metabolomics

Abiotic stress, encompassing environmental challenges like drought, salinity, extreme temperatures, and heavy metal toxicity, profoundly impacts plant growth and productivity. These stresses disrupt various physiological and biochemical processes in plants [51]. Metabolomics, the comprehensive study of metabolites in plant systems, plays a vital role in understanding how plants adapt to these stressors at a molecular level. By identifying key metabolites involved in stress responses, metabolomics provides insights into the biochemical mechanisms that underpin plant resilience and survival under adverse conditions [52].

#### 3.5.1. Abiotic Stress Responses

Plants respond to abiotic stresses by undergoing various metabolic adjustments to maintain homeostasis. A key response to abiotic stress is the buildup of osmoprotectants like proline and glycine betaine. These compounds help to stabilize proteins and cellular structures under osmotic stress [53]. For instance, under drought conditions, plants often accumulate proline, a compatible solute that helps protect the plant’s cellular integrity [54]. In addition to osmoprotectants, plants produce a range of antioxidants, including flavonoids, which mitigate oxidative stress induced by environmental stressors. These antioxidants scavenge reactive oxygen species (ROS) that can cause cellular damage [55]. The metabolic shifts that occur in response to drought, salinity, and temperature extremes are crucial for maintaining plant functionality and ensuring survival in harsh environments [51].

For instance, drought stress induces significant metabolic adjustments in plants, particularly in amino acid metabolism. Under water-limiting conditions, the accumulation of certain amino acids, such as asparagine, glutamine, and glutamate, is reduced, while branched-chain amino acids (BCAAs) like isoleucine, leucine, and valine are significantly increased. This metabolic shift reflects an adaptive strategy to conserve energy and maintain cellular functions, thereby enhancing plant survival under drought stress [56,57]. Salinity stress, however, primarily affects ion balance and osmotic potential within plant cells. To cope with the increased ionic concentration in the soil, plants accumulate metabolites involved in ion transport, such as betaine, a quaternary ammonium compound. Betaine helps plants maintain cellular function under saline conditions by stabilizing proteins and promoting osmotic adjustment [58]. Salinity stress also leads to shifts in primary metabolic pathways, with plants adjusting their energy production and cellular signaling processes to mitigate the effects of high salt levels [58]. Extreme temperature stress, whether from high or low temperatures, triggers significant metabolic responses in plants. Heat shock proteins (HSPs) are produced in response to elevated temperatures, and they assist in protein folding and stabilization, preventing protein denaturation [59]. Meanwhile, secondary metabolites like flavonoids and other antioxidants accumulate as a response to heat stress. These compounds act as protective agents by scavenging ROS, generated in excess under high-temperature conditions. The production of antioxidants is crucial for safeguarding plant cells against oxidative damage induced by thermal stress [60].

#### 3.5.2. Stress Adaptation Mechanisms

Metabolites involved in stress responses often influence essential physiological processes such as photosynthesis and respiration. By examining these metabolic adaptations, researchers can develop crops better suited to withstand environmental changes, particularly those arising from climate change. For example, plants often adjust their photosynthetic and respiratory processes to optimize energy use during stress. Alterations in carbohydrate metabolism help meet the plant’s energy demands, supporting its survival under challenging conditions [61]. Combining metabolomics with other “omics” technologies, such as transcriptomics and proteomics, offers a more comprehensive view of how plants sense and respond to abiotic stress. This comprehensive approach allows scientists to connect changes in metabolites to gene expression and protein activity, yielding deeper insights into the regulatory networks that govern plant resilience [62]. Through pathway analysis, key metabolic pathways that are activated or repressed during stress can be identified, enhancing our understanding of plant adaptive strategies. As technologies in this field continue to advance, they enhance our understanding of plant biology, which in turn aids in improving crop management and developing effective resistance strategies [63].

#### 3.5.3. Applications in Plant Science

Metabolomics is increasingly being utilized to study plant responses to biotic and abiotic stresses. By analyzing the changes in metabolite profiles under different stress conditions, researchers gain valuable information that can be applied to crop improvement efforts [64]. Exploring how plants metabolically respond to stress can aid in creating resilient crop varieties and inform agricultural practices, ultimately boosting crop productivity and sustainability amid environmental challenges.

#### 3.5.4. Challenges in Metabolomics

Although metabolomics has made considerable progress, several challenges still limit its full potential, especially in the context of plant stress biology. One of the primary challenges is the qualification and quantification of specialized metabolites. Many metabolites, especially those produced in response to biotic and abiotic stressors, lack standardized reference materials [64]. This makes their identification and functional analysis more complex. The lack of comprehensive reference materials presents a significant challenge in accurately quantifying and understanding the functional roles of specific metabolites. To address this issue, researchers are utilizing advanced analytical approaches that combine retention time analysis, exact mass measurements, and UV spectral data. These integrated methods offer detailed structural insights into unidentified metabolites, facilitating more accurate identification and characterization [65].

#### 3.5.5. Applications in Crop Improvement

The insights from metabolomic studies have profound implications for agricultural practices and crop improvement. By pinpointing essential metabolites linked to stress tolerance, researchers can guide breeding efforts to create crop varieties that are better equipped to withstand environmental stresses. To demonstrate, selecting metabolites that play crucial roles in osmotic regulation, antioxidant defense, or secondary metabolism could lead to the development of crops that thrive under challenging conditions such as drought, salinity, or pathogen pressure [66].

Moreover, metabolomics can inform agronomic practices by guiding decisions on optimizing irrigation, fertilization, and pest control strategies based on the metabolic responses of crops to stress. This enables farmers to implement more targeted and efficient agricultural practices that improve crop yield and sustainability. In addition, understanding the metabolic pathways involved in stress responses opens up the possibility of applying biotechnology to enhance crop stress tolerance. Through genetic engineering or CRISPR-based technologies, specific pathways can be targeted to increase stress resistance, thus improving crop resilience at a genetic level [66,67].

#### 3.5.6. Future Directions

The future of metabolomics in plant science holds tremendous promise, particularly with regard to its integration with other omics technologies. By integrating metabolomics with genomics and proteomics, researchers can gain a more comprehensive understanding of plant stress responses. This integrated approach will help identify regulatory networks and metabolic pathways involved in plant defense mechanisms, facilitating more precise breeding and management strategies [68]. Technological advancements are also a key component of the future of metabolomics. Continued improvements in analytical methods and instrumentation, such as higher resolution and sensitivity in mass spectrometry and better computational tools for data analysis, will expand the capability to study complex metabolic networks and their interactions with environmental factors. These advances will make it possible to identify more metabolites, quantify their abundance more accurately, and understand their dynamic roles in plant stress responses [69].

Finally, translating laboratory findings into field applications remains a crucial challenge. While much of the metabolomics research has been conducted under controlled laboratory conditions, applying these insights to real-world agricultural settings will be key to addressing global food security challenges. Developing strategies to monitor crop health and stress responses in real time, for example, through portable metabolomic devices or on-field sensors, will be essential for ensuring that metabolomic insights can be applied in practical farming environments [70].

### 3.6. Metabolomic Insights into Trehalose-Mediated Resilience Against Biotic and Abiotic Stress

Recent breakthroughs in metabolomics have highlighted the critical role of trehalose in boosting plant resistance to both biotic and abiotic stresses, especially salinity. A study on rice varieties Giza 177 and Giza 178 revealed that applying trehalose effectively reduced the negative impacts of salinity, improving growth and preserving photosynthetic efficiency. In saline environments, trehalose not only promoted the accumulation of important metabolites like total soluble sugars and proline but also triggered the activation of vital antioxidant enzymes, such as superoxide dismutase (SOD) and catalase (CAT), which play a key role in mitigating oxidative stress. The findings indicated that Giza 178 exhibited greater tolerance to salinity compared to Giza 177, highlighting the genetic variability in stress responses among rice varieties. This research underscores the potential of trehalose as a strategic tool in crop management, providing insights into the metabolic pathways that can be harnessed to develop stress-resistant cultivars. By integrating metabolomic profiles with physiological responses, researchers can better understand the mechanisms underlying plant resilience, paving the way for innovative agricultural practices in environments challenged by salinity and other stressors [71].

## 4. Ionomics and Interomics Against Plant Biotic and Abiotic Stress

In plants, ionomes help with a number of tasks, such as regulating the functionalities of the plant genome. The development of ionomics has been greatly aided by a variety of genetic mapping techniques [72]. The Purdue Ionomics Information Management System (PIIMS) serves as a comprehensive database encompassing the ionic characteristics of plants [73]. Advances in ionomics are being used in genome-wide association analysis of genetic variation within plant species. Ionomics research across diverse crops generates valuable insights into their functions and facilitates the identification of resistant genotypes. This leads to a contribution of a deeper understanding of host–pathogen interactions [74]. High-throughput elemental analysis technologies must be used in ionomics, and they must be integrated with genetic and bioinformatics techniques [75]. This helps in evaluating the abiotic stress tolerance in the plant system, which is a genetically complex process that requires numerous signaling pathway components, as opposed to biotic stress, which is governed by monogenic traits [76].

In order to drive phenotypic fitness-related features, ionomics also investigates the potential roles of all atoms constituting the plant’s organismal structures [77]. These elemental proportions can be adjusted within the limits of stoichiometric homeostasis and may vary in response to environmental stressors. Furthermore, various studies demonstrated that plants’ abiotic stress under heavy metal in silicon had lower levels of ROS, a sign of an improved stress response. Further, the role and mechanism of exogenous silicon in enhancing tomato salt tolerance revealed that silicon effectively reduced Na and Cl concentrations in the roots, stems, and leaves while maintaining the efficient transport of essential nutrients from roots to shoots [78]. Extensively, the inductively coupled plasma–mass spectrometry (ICP-MS) approach has been used to completely analyze the ionomic profile of dicot plants, such as *A. thaliana*. This work contributes to the full genomic analysis of the plant, encompassing all QTLs (qualitative trait loci) that control the activity of the genes linked to the ionome investigation [79]. The dynamic aspect of omics-based stress tolerance is ionomics, which entails a thorough analysis of plant elemental profiles and offers insights into metal toxicity and nutrient variations during both abiotic and biotic stress [8]. This approach has also tracked the exclusion of ions under unfavorable environmental conditions [1]. Furthermore, it can be utilized to rectify the negative effects of high-salinity-induced osmotic stress and excessive Na+ concentration on root function [80]. Furthermore, the most suitable and promising methods for ionomic and environmental investigations of plants have also been reviewed using atomic absorption spectrometry (AAS) and ICP-MS [81].

Interomics is another interdisciplinary approach that combines multiple omics technologies, including genomics, transcriptomics, proteomics, metabolomics, and epigenomics, to achieve a comprehensive understanding of plant biological systems [82]. By utilizing multi-omics approaches, researchers can unravel the intricate signaling pathways and alterations in gene expression that occur during stress conditions, providing insights into how plants adapt and respond to environmental challenges [83]. This integrated perspective not only enhances our understanding of stress tolerance mechanisms but also facilitates the development of resilient crop varieties [3].

## 5. Phenomics Against Abiotic and Biotic Stress

Phenomics is a crucial area in plant research that can be used to study the large-scale responses of plants to various stresses. Biotic stress, induced by living organisms such as pathogens, pests, and weeds, poses a major threat to agricultural productivity, with diseases and pest infestations contributing to considerable yield losses worldwide [84]. Phenomics technologies, such as high-throughput phenotyping (HTP), play a pivotal role in identifying resistance traits in plants [85]. High-throughput phenotyping platforms enable the evaluation of salinity tolerance by measuring traits such as ion accumulation, growth inhibition, and osmotic regulation, while also analyzing nutrient uptake efficiency through advanced imaging technologies [86]. Abiotic stressors, such as climate change, are becoming increasingly prevalent, affecting agricultural productivity by affecting plant growth, development, and yield [87]. Phenomics approaches are essential for assessing plant tolerance and adaptation mechanisms to these stresses. The key applications include drought stress tolerance, salinity and nutrient stress, and temperature stress [88]. The incorporation of advanced technologies in phenomics has transformed stress analysis, with imaging techniques such as thermal, hyperspectral, and fluorescence imaging enabling accurate monitoring of plant responses [89]. Further, drones and satellite imaging facilitate large-scale phenotyping in field conditions, providing real-time data on stress impacts across agricultural landscapes [90]. Phenomics holds great promise in tackling global food security issues; however, challenges such as the high cost of phenotyping platforms and the need for standardized methodologies across experiments persist. Future research should aim at developing affordable and scalable phenomics solutions to support resource-constrained regions [91].

## 6. Decoding Plant Stress Responses Through Gene Regulation

Plants are frequently exposed to many environmental stressors, like drought, high temperatures, and salinity, as well as biotic factors like viruses, bacteria, insects, etc. These stressors impact the growth of plants and agricultural productivity [92,93]. To manage these challenges, plants utilize intricate regulatory systems that govern gene expression through multiple layers, including transcriptional, post-transcriptional, and epigenetic mechanisms. Advances in multi-omics technologies have provided deeper insights into these regulatory networks, facilitating the development of stress-tolerant crops [9,92].

Gene expression during stress is primarily governed by TFs that bind to specific DNA sequences in the promoters of target genes. TFs such as DREB and CBF are critical in activating pathways that respond to stress [94]. For instance, under cold stress, the DREB1/CBF factors induce the expression of genes, which help plants survive harsh conditions [95]. Studies using transcriptomics have shown that the activity of these TFs is closely related to the type of stress, offering potential targets for genetic engineering aimed at improving stress tolerance [92]. In addition to transcriptional regulation, post-transcriptional processes like RNA splicing, stability, and editing significantly influence stress responses. Alternative splicing generates diverse protein isoforms from a single gene, allowing plants to adapt flexibly to different stress conditions. This mechanism has been documented in stress-responsive genes like SR45, which produces isoforms adapted to distinct functions in response to abiotic stress [96].

Epigenetic changes such as histone modifications, DNA methylation, and chromatin remodeling also play a vital role in regulating gene expression under stress. DNA methylation at gene promoter regions can either activate or repress stress-responsive genes [97]. Additionally, plants can retain stress-induced epigenetic marks across generations, a phenomenon termed transgenerational epigenetic memory, which enables offspring to better cope with similar stress conditions [98].

ROS, such as hydrogen peroxide (H_2_O_2_), serve as early signals in plant stress responses. These molecules trigger signaling cascades that activate stress-responsive genes. Plant hormones like salicylic acid (SA), abscisic acid (ABA), and jasmonic acid (JA) interact with ROS to modulate stress responses [99]. For example, SA plays a pivotal role in localized and systemic defenses, coordinating the activation of genes involved in pathogen resistance and stress adaptation. The interplay between ROS and hormonal signaling ensures a well-coordinated response to environmental stresses [100].

Recent technological advancements have made it possible to monitor plant stress responses in real time. For example, nano sensors have been created to detect essential signaling molecules like H_2_O_2_ and SA at the same time [101]. These tools provide insights into the temporal dynamics of stress signaling and enable the early identification of stress, even before visible symptoms appear. Such innovations are crucial for understanding plant stress physiology and devising strategies to mitigate yield losses [101].

## 7. Gene Regulation Under Abiotic Stress

**Figure 2:**
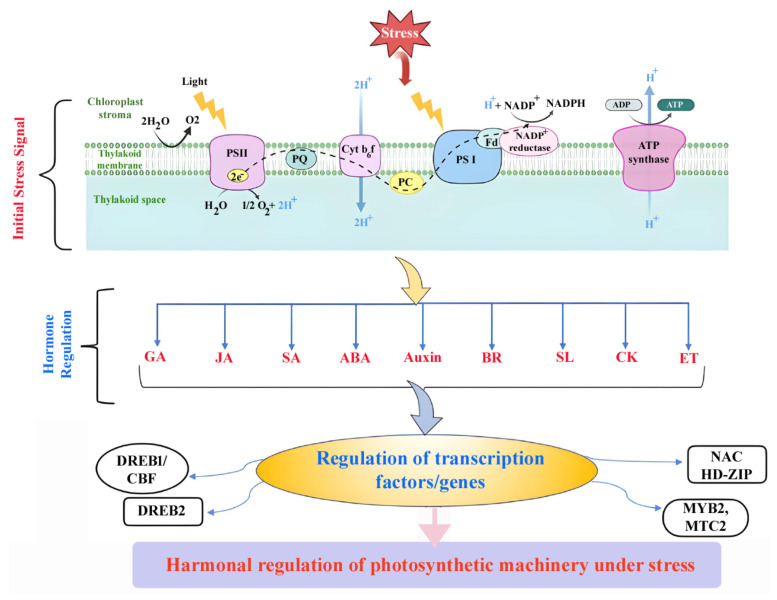
Gene regulation against abiotic/biotic stresses.

Abiotic stresses, including drought, extreme temperatures, and salinity, present substantial challenges to plant survival and productivity. To overcome these stresses, plants have developed advanced mechanisms for detection and adaptation. One crucial approach is the regulation of gene expression, enabling plants to activate or deactivate specific genes in response to stress signals [9,102,103]. Plants sense environmental stress through specialized receptors and sensor proteins. These signals are processed through complex signaling pathways, which activate specific TFs such as DREB (Drought-Responsive Element Binding), MYB, and NAC (Figure 2) [104]. These TFs bind to promoter regions of stress-responsive genes, influencing their transcription. For instance, under drought conditions, DREB2A binds to drought-responsive elements (DREs) in the promoters of dehydration-responsive genes, initiating their expression of many water-stress-inducible genes [105]. Epigenetic modifications, including DNA methylation and histone modification, play a crucial role in regulating gene expression under abiotic stress conditions. These modifications alter chromatin structure and gene accessibility, thereby influencing transcription [106]. For example, histone acetylation, mediated by histone acetyltransferases (HATs), and deacetylation, controlled by histone deacetylases (HDACs), undergo dynamic shifts in response to stress. Enhanced histone acetylation at the loci of stress-responsive genes is linked to elevated gene expression and improved stress tolerance [107].

Proteomics and metabolomics studies have further advanced our understanding of plant responses to abiotic stress at the protein and metabolite levels [108]. Proteomic analyses have highlighted changes in levels of stress-associated proteins, such as antioxidants and chaperones, which help protect plants under adverse conditions. Metabolomics has identified key metabolites like proline and glycine betaine, which accumulate under osmotic stress to maintain cellular stability [109]. The advent of multi-omics approaches has revolutionized the study of gene regulation under abiotic stress. Technologies like RNA sequencing (RNA-seq) and chromatin immunoprecipitation sequencing (ChIP-seq) allow comprehensive genome-wide analysis of gene expression and epigenetic modifications. These tools have illuminated the intricate regulatory networks governing stress responses and identified key components for potential genetic and epigenetic interventions [9,92].

Additionally, new bioinformatics tools and computational methods have streamlined the integration and analysis of multi-omics data. Machine learning techniques are increasingly employed to model regulatory networks and pinpoint essential regulatory elements in stress response pathways. Such innovations have expedited the discovery of stress-responsive genes and mechanisms, facilitating the development of resilient crop varieties [110]. Gene regulation under abiotic stress is governed by a sophisticated interplay of signaling pathways, TFs, and epigenetic mechanisms [110]. Multi-omics integration has significantly deepened our understanding of these processes, offering promising strategies to enhance crop resilience. Technological progress continues to drive innovations in studying and engineering stress tolerance; it is vital for improving agricultural productivity and food security in an era of global climate change.

## 8. Gene Regulation in Biotic Stress

Recent research underscores the interconnected nature of biotic and abiotic stress responses in plants, with DREBs (Dehydration-Responsive Element-Binding proteins) serving a central role. Plants in natural environments rarely experience a single stress; instead, they are often subjected to multiple, simultaneous challenges. For instance, inoculation of *A. thaliana* with growth-promoting rhizobacteria improved resistance against *Erwinia carotovora* and dehydration stress, revealing a link between disease resistance and drought tolerance [111]. Similar findings were observed in *Castanea sativa*, where abiotic stress induced the production of the antifungal protein cystatin [112]. Moreover, studies have demonstrated cross-communication between ABA-independent dehydration-responsive pathways involving *DREB2A* and *adr1* (activated disease resistance 1) signaling [113]. The overexpression of ADR1 significantly enhanced drought tolerance, though it did not improve resistance to thermal or salt stress. Investigations into *adr1* mutants showed a salicylic acid (SA)-dependent increase in *DREB2A* expression mediated by reactive oxygen intermediates (ROIs), while other stress-associated genes, such as *DREB1A*, *rd29A*, and *rd22*, showed no changes [113]. Additionally, microarray analyses indicated that ADR1 activation led to the upregulation of numerous drought-responsive genes, providing further evidence of the overlap between biotic and abiotic stress pathways. These findings highlight the complexity of stress responses in plants and the regulatory roles of DREB proteins in these processes [113].

### 8.1. Insect-Induced Promoters in Response to Biotic Stress

In potatoes, the proteinase inhibitor II *(pinII)* gene is activated in response to insect attacks or significant wounding. Similarly, in transgenic *Arabidopsis* plants with the GUS gene controlled by the *pinII* promoter, expression is induced by insect damage or wounds [114]. The *pinII* promoter is a versatile tool for activating defense-related genes across multiple plant species, making it a valuable candidate for biotic stress management [115]. For instance, transgenic peanut (*Arachis hypogaea*) plants with the *Cry1EC* transgene under the inducible *PR1-a* promoter demonstrate increased resistance to *Spodoptera litura* [116]. The *PR1-a* promoter is especially effective in creating transgenics targeting aphid resistance due to its activation during aphid attacks. Broccoli transgenics expressing the *Cry1Ab* gene regulated by the *PR1-a* promoter also exhibit resistance to the diamondback moth, *Plutella xylostella* [117].

### 8.2. Promoters Induced by Pathogen Infections

Pathogens such as viruses, bacteria, and fungi pose serious threats to global agriculture. Plants counter these threats by producing pathogen-responsive proteins, including PR proteins and antiviral agents. Transgenic expression of such proteins has improved resistance in crops [118]. For example, the *Phenylalanine Ammonia-Lyase (PAL1)* promoter is activated during bacterial infection caused by *P. syringae* [119]. Promoters of pathogen-responsive genes often contain cis-regulatory elements like W-boxes, elicitor-responsive motifs, TC-rich repeats, and TCA elements. For example, in parsley, the *CMPG1* gene is quickly induced upon pathogen exposure, with its promoter relying on such regulatory sequences for activation [120].

### 8.3. Role of LncRNAs in Plant Defense Mechanisms Against Biotic Stress

A study on the disease-responsive long non-coding RNAs (lncRNAs) in rice, particularly in response to Rice Stripe Virus and Rice Black-Streaked Dwarf Virus, revealed 21 upregulated lncRNAs. These lncRNAs were linked to over 1000 co-regulated genes involved in processes such as transcription regulation, plant hormone signaling, phenylpropanoid biosynthesis, and plant–pathogen interactions [121]. One lncRNA, known as Salicylic Acid Biogenesis Controller 1 (*SABC1*), is involved in regulating salicylic acid production, which helps balance plant growth and immunity. *SABC1* interacts with the CURLY LEAF (CLF) protein, a component of the polycomb repressive complex 2 (PRC2), to inhibit the *NAC3* gene through H3K27me3 catalysis. While *SABC1* typically downregulates salicylic acid production and immunity under normal conditions, its expression decreases during pathogen infection, reducing repression and boosting the plant’s immune response [122]. In conclusion, plants employ intricate regulatory networks, involving TFs, microRNAs, and epigenetic modifications, to adapt to biotic stress and respond to pathogen attacks and environmental challenges.

## 9. Transcriptional Networks in Response to Abiotic and Biotic Stress

Transcriptional networks regulate the effects of biotic and abiotic stressors on plants by modulating gene expression through TFs and their interactions [123,124]. The evolutionary importance of polyploidy in forming crop genomes has been made clear by the quick next-generation sequencing (NGS) developments. Network-based approaches that integrate diverse technologies, such as RNA-seq and microarrays, are essential for overcoming the challenges in understanding the roles and variations in the temporal regulation of abiotic stress pathways that influence yield [124]. Two major players in the crosstalk between signaling pathways are TFs and kinases. Significant roles are played by signaling pathways regulated by—-SA, (ABA), ethylene (ET), ROS and JA in the interaction between abiotic and biotic stress signals [125]. While JA, ET, and SA are significant in biotic stress signaling, the main function of ABA is to respond to abiotic stressors such as osmotic stress, low temperature, and drought [125].

### 9.1. AREB/ABF TFs Regulate Gene Expression in an ABA-Dependent Manner

ABRE [U1] (Abscisic Acid-Responsive Element) is a conserved cis-element found in promoter domains of ABA-responsive genes that regulate gene expression. It has been reported that a functional promoter to express ABA-responsive genes requires either many ABREs or a combination of an ABRE and a coupling element (CE) [126]. The ABRE serves as the primary cis-regulatory element for ABA-responsive gene expression, and extensive molecular and comprehensive studies have shown that ABA modulates the expression of numerous genes under osmotic stress conditions [127,128]. Similarly, RCAR/PYR/PYL ABA receptors, group A PP2Cs, and SnRK2 have been shown to modulate the ABA signaling pathway, which includes AREB/ABFs in terrestrial plants [129]. In ABA-dependent signaling networks, ABFs/AREB is essential for SnRK2s’ phosphorylation [130]. Recent findings indicate that group A PP2Cs, key regulators of intrinsic desiccation resistance, emerged early in land plants, including the moss *Physcomitrella patens* [131]. Stress tolerance can also be improved by targeting perception and signaling components such as PYL4 [127,132,133].

### 9.2. DREB1/CBF Regulon’s Response to Cold Stress

Gene Chip microarrays and cDNA have been used to identify over 40 target *DREB1/CBF* genes. In their promoters, the Dehydration-Responsive Element/C-Repeat (*DRE/CRT*) or similar core motifs were present in the majority of those target genes [134]. Phospholipase C, TFs, RNA-binding proteins, desaturases, sugar transport proteins involved in the metabolism of carbohydrates, KIN (cold-inducible) proteins, osmoprotectant biosynthesis proteins, phospholipase C, and protease inhibitors are among the target proteins [134]. The observed stress tolerance in transgenic overexpressor lines is attributed to the activity of target proteins, which are known to respond to stress. Particularly, under controlled conditions, transgenic plants overexpressing *DREB1A/CBF3* accumulated osmoprotectants such as proline and various sugars [135]. Under optimal growth conditions, overexpression of *DREB1/CBF* led to notable growth retardation. To mitigate the negative impact on growth and development, the stress-inducible *rd29a* promoter was employed for *DREB1A* overexpression, rather than the strong constitutive *CaMV 35S* promoter [136].

### 9.3. Improved Drought Resistance Through DREB2 TFs: Heat-Responsive and Osmotic Gene Expression

The domain analysis of *DREB2A* using *Arabidopsis* protoplasts showed that deletion of the central region results in constitutive activation of *DREB2A* (*DREB2Aca*), indicating the presence of a negative regulatory domain [105]. Then, DREB2Aca overexpression increased drought tolerance, upregulated stress-inducible genes, and caused growth abnormalities [105]. Conversely, stress-induced *DREB2ca* overexpression enhanced drought tolerance in soybeans and *Arabidopsis* without causing growth abnormalities [137]. The regulation of *DREB2A* is controlled by its negative regulatory domain (NRD) region. As mentioned earlier, overexpression of *DREB1A* enhances transgenic plants’ resistance to freezing and dehydration stress. In contrast, overexpression of *DREB2Aca* improves resistance to dehydration stress but only slightly enhances tolerance to freezing stress in transgenic plants [137].

The expression of genes linked to glucose metabolism differs greatly between *DREB1A* and *DREB2A* transgenic plants, despite the microarray studies suggesting that the downstream gene products of *DREB1A* and *DREB2A* have similar roles [137]. Cold and dehydration stress trigger significant changes in the expression of genes related to sucrose metabolism, sugar alcohol synthesis, and starch degradation, leading to the accumulation of various sugars and alcohols in plants [138]. Overexpression of *DREB1A* induced similar metabolic changes, enhancing the resistance of transgenic plants to freezing and dehydration stress. However, *DREB2Aca* overexpression did not result in an increase in the concentration of these metabolites in transgenic plants [138].

### 9.4. Drought-Responsive Gene Expression via NAC TFs to Enhance Drought Tolerance

Overexpression of rice SNAC genes, *OsNAC6*/*SNAC2*, and *OsNAC5*, as well as *Arabidopsis* SNAC genes, *rd26*, and *ATAF1*, responding to stress can enhance tolerance to high-salt stress and/or drought [139]. Plant-specific TFs include ATAF, CUC, and NAM proteins. Over 100 NAC genes have been identified in both rice and Arabidopsis. NAC TFs play essential roles in stress responses and developmental processes of the crops. Genes within the SNAC group are capable of binding to the NAC recognition sequence (NACR), which includes the CACG core, and are crucial for regulating tolerance to environmental stresses [140]. Stress tolerance can be effectively induced without NAC’s growth-inhibiting effects by using rice stress-responsive *OsHox24*, or *OsNAC6*, LIP9 promoters to overexpress NACs [141,142].

Recent studies suggest that overexpressing SNACs, such as *SNAC1* and *OsNAC10*, using the root-specific promoter RCc3, can enhance rice’s ability to withstand abiotic stress under field conditions [143]. These findings indicate that SNACs are crucial in regulating abiotic stress responses and tolerance. Overexpressing SNACs with suitable field promoters can improve stress resilience. By utilizing various tissue- or organ-specific as well as drought-responsive promoters identified for roots and stomata, it may be possible to precisely control the expression of drought-related factors that affect growth, at the optimal time and location [141].

### 9.5. Crosstalk Between Biotic and Abiotic Stress

TFs and kinases are crucial in facilitating crosstalk between signaling pathways for stress responses (Table 1). The hormonal crosstalk includes ABA, JA, SA, ET. ABA regulates abiotic stress responses like drought, low temperature, and osmotic stress [125]. SA, JA, and ET genes are predominantly involved in biotic stress [U4] signaling, where ubiquitin 4 genes are involved [144]. ABA plays an antagonistic role by repressing the expression of JA/ET-mediated defense genes, suggesting a conflict between these pathways. Key regulators include RD26 (an NAC transcription factor), which is triggered by ABA, JA, H_2_O_2_, pathogens, drought, and salinity, and *AtMYC2* (a bHLH transcription factor), which stimulates ABA-mediated drought stress signaling but negatively regulates JA/ET defense pathways. RD26 regulates ROS detoxification, defense responses, and senescence, acting as a convergence point for various stress signals [145]. In Arabidopsis, kinase cascades like MEKK1-MKK2-MPK4/MPK6 are activated under cold and salt stress, while MEKK1-MKK4/MKK5-MPK3/MPK6 regulates pathogen defense through WRKY22 and WRKY29. MPK3/MPK6 is primarily involved in oxidative stress and hormone signaling pathways, where ubiquitin 5 genes are involved [U5] (Table 1) [146]. Then, ROS function as key signaling molecules in both ABA signaling and disease resistance, regulated by kinases such as *OXI1*, *ANP1*, and *AtNDPK2*, enhancing ROS scavenging and stress tolerance. Calcium-dependent protein kinases (CDPKs) mediate stress responses via SA, ET, JA, and ABA signaling pathways in tobacco plants [84,147].

## 10. Challenges Involved in Detection of Both Abiotic and Biotic Stressors

Improving crop productivity is a key challenge for a country’s economy. A major factor that hampers agricultural yields is plant stress, which can be broadly classified into biotic and abiotic types [160]. Abiotic stress arises from environmental factors such as temperature, nutrient imbalances, soil pH, and salinity, while biotic stress is caused by biological agents such as pests, pathogens and weeds. The identification of these stresses in early stages will increase the crop yield by minimizing the over-usage of pesticides and the unwanted use of fertilizers [161]. Traditionally, stress detection relied on observing changes in plant morphology, involving visual inspection followed by isolation of suspected pathogens and performing bioassays. However, traditional techniques face significant challenges, including being time-consuming and impractical for analyzing large agricultural fields [162,163]. In recent years, with the advancement in technology such as thermal imaging, hyperspectral and multispectral imaging, and positron emission tomography, remote sensing has been used to detect and analyze both abiotic and biotic stresses with greater precision and efficiency even for larger agricultural fields [162,163]. Combining these techniques with AI and machine learning enhances the processing of complex datasets, leading to more accurate results [164]. Despite recent advancements in stress detection, several challenges persist, including the need for vast amounts of data to make accurate predictions, sensor limitations that can impact data accuracy under varying environmental conditions, and the inability to identify the specific types of abiotic or biotic stresses affecting plants. Additionally, it struggles to distinguish between molecular symptoms with similar characteristics [162,163]. When a plant is simultaneously exposed to both abiotic and biotic stressors, these methods may struggle to identify and differentiate the root causes, resulting in inaccurate conclusions. These challenges can be mitigated by employing complementary approaches, such as omics studies, including metabolomics and proteomics, which provide deeper insights. Then, metabolomics provides insights into the biochemical pathways and metabolic alterations associated with stress, while proteomics reveals protein interactions and changes in protein expression during stress [1]. This technique enables the identification of molecules involved in biochemical pathways and protein interactions during various stress types. Being based on molecular detection, it can effectively differentiate between biotic and abiotic stresses affecting the plant, resulting in more precise and reliable data [1]. Hence, integrating this complementary approach with imaging technologies can substantially improve crop productivity.

## 11. Strategies for Improving Stress Tolerance

Biotic and abiotic stresses have a profound effect on plant growth and crop productivity, representing a major challenge to global agriculture [165]. While plants can naturally tolerate stress through physiological responses, biochemical adjustments, osmotic regulation, and the production of secondary metabolites, these inherent defenses often fall short under extreme conditions. Therefore, further enhancement of these mechanisms could improve stress tolerance and increase plant yield [166]. Several advanced strategies are utilized to improve the performance of crops under stress, including the use of plant growth-promoting rhizobacteria (PGPR), genome-editing technologies like clustered regularly interspaced short palindromic repeats (CRISPR/Cas9), and tools such as NGS to identify stress-responsive genes [166,167,168]. PGPR are beneficial soil microorganisms that play a significant role in balancing abiotic stress by promoting root growth and enhancing nutrients and uptake of water. They achieve this through the synthesis of phytohormones such as JA, indole-3-acetic acid (IAA), salicylic acid, and gibberellins, which stimulate lateral root formation and root elongation [168]. Additionally, PGPR activates the plant’s antioxidant defense system, which protects cell membranes from damage caused by reactive oxygen species (ROS) during stress [169]. Stress leads to elevated ethylene levels in plants, a hormone that inhibits root and shoot growth. This effect is mitigated by plant growth-promoting rhizobacteria (PGPR) through the action of the enzyme ACC deaminase, which degrades 1-aminocyclopropane-1-carboxylic acid (ACC), the precursor to ethylene, thus reducing ethylene toxicity [169]. Additionally, PGPR produces volatile organic compounds like 2,3-butanediol, which assist in regulating stomatal opening, thereby enhancing water-use efficiency under stress conditions [169]. The CRISPR/Cas9 genome-editing tool has emerged as an advanced tool for enhancing both abiotic and biotic stress tolerance in plants by enabling genetic modifications. This technique involves identifying stress-responsive genes, designing a single guide RNA complementary to the target gene, and introducing the guide RNA and Cas9 enzyme into plant cells through delivery systems such as *Agrobacterium tumefaciens*-mediated transformation or particle bombardment [166]. The Cas9 protein generates precise double-strand breaks at specific locations in the DNA, which are then repaired by the plant’s natural repair mechanisms. These repairs can occur through non-homologous end joining (NHEJ), which disrupts undesirable genes, or homology-directed repair (HDR), which introduces beneficial genes [166]. Omics studies offer valuable insights into identifying stress-responsive genes. By integrating this approach with CRISPR/Cas9 technology and next-generation sequencing, it is possible to develop stress-resilient crops, thereby enhancing agricultural productivity.

## 12. Role of Secondary Metabolites in Mitigating Stress Response

Secondary metabolites are organic compounds produced by plants that play a crucial role in plant defense mechanisms against biotic and abiotic stresses [170]. These metabolites include terpenoids, flavonoids, alkaloids, and phenolics, which contribute significantly to plant resilience and survival [171]. Secondary metabolites serve as a first line of defense against pathogens, inhibiting the growth of bacteria, fungi, and viruses. They can alter cellular permeability and disrupt pathogen metabolism, leading to reduced pathogen proliferation. Stress signaling is another mechanism in which secondary metabolites activate plant defense pathways [170,171]. They can enhance the expression of defense-related genes through interactions with TFs, strengthening the plant’s immune response. Some secondary metabolites attract beneficial organisms, enhancing plant survival by promoting ecological interactions. Secondary metabolites produced by plants play a crucial role in helping them withstand abiotic stresses, including drought, salinity, and extreme temperatures [172]. These biometabolites act as osmoprotectants, maintaining the stability of proteins and cellular structures under osmotic stress, while also serving as antioxidants by neutralizing reactive oxygen species (ROS) produced during stressful conditions. They function as osmoprotectants, stabilizing proteins and cellular structures under osmotic stress, and antioxidants, scavenging reactive oxygen species (ROS) generated during stress conditions [170]. Further, terpenoids are involved in direct antimicrobial activity and can attract beneficial organisms. In addition, flavonoids are known for their antioxidant properties and play a crucial role in UV protection and signaling during stress responses [170]. Furthermore, alkaloids, often toxic to herbivores and pathogens, deter feeding and inhibit microbial growth. Ultimately, phenolics play a key role in providing structural defenses and facilitating signaling pathways during stress responses. Finally, phenolics are crucial for structural defenses and signaling pathways during stress responses [170].

Secondary metabolites are bioactive molecules that not only trigger plant defense responses when they encounter stress. They also activate specific pathways, which in turn trigger the expression of genes involved in the plant’s defense responses and activate specific pathways, leading to the expression of defense-related genes [170]. Bioactive metabolites such as flavonoids and phenolics can induce these genes through their interaction with TFs, which can regulate gene expression even under plant stress conditions [173]. The WRKY family of TFs plays a critical role in this process by detecting stress signals and activating downstream genes that are involved in defense responses, including those that regulate the production of secondary metabolites. The WRKY family of TFs is particularly significant in this context, as they recognize stress signals and activate downstream genes involved in defense mechanisms, including those responsible for synthesizing additional secondary metabolites [172].

Plants produce phytoalexins, antimicrobial secondary metabolites, in response to pathogen attack or environmental stress [174]. The synthesis of these compounds is often regulated by specific TFs that respond to stress signals [172,174,175]. Secondary metabolites also facilitate crosstalk between different signaling pathways, enhancing the plant’s ability to respond to both biotic and abiotic stresses [172]. They also play a role in “stress memory,” where previous exposure to stress strengthens future responses through epigenetic modifications driven by the accumulation of these metabolites. Another crucial feature of secondary metabolites is their antioxidant activity, which helps reduce oxidative damage induced by environmental stresses. Compounds like phenolics and flavonoids protect cellular components and maintain metabolic function under stress conditions [172,176].

## 13. Role of Bio-Based Products in Stress Tolerance

Bio-based products, derived from plants and beneficial microbes, are increasingly recognized for their role in enhancing stress tolerance in agriculture. These compounds serve as bio-stimulants and biocontrol agents, addressing both biotic and abiotic stresses that significantly impact crop yield and quality [177]. Bio-based compounds activate various physiological and biochemical pathways in plants, improving their resilience to stress. For example, brassinosteroids enhance salt tolerance in potatoes by regulating gene expression related to stress responses [178]. Beneficial microbes like *Trichoderma hertzian* and *Bacillus subtilis* can improve plant growth and mitigate heavy metal toxicity in contaminated soils [179]. Certain plant cultivars accumulate bioactive compounds that enhance their defensive capabilities against pests. Bio-stimulants, such as microbial inoculants applied to seeds, help plants cope with stress by activating stress tolerance pathways and boosting carbohydrate production, which improves overall plant health. However, more research is required to better understand the molecular processes at play and their long-term impact on soil health [180]. Overall, bio-based products show great potential for supporting sustainable agriculture by strengthening plants’ ability to handle stress through various biological mechanisms [176,178].

Bio-based products in agriculture offer numerous benefits for improving plant stress tolerance, addressing both biotic and abiotic stress [181]. These products, derived from plants, algae, and beneficial microbes, are becoming sustainable alternatives to traditional agrochemicals. They promote plant growth, enhance nutrient uptake, increase photosynthetic efficiency, and stimulate root development, thereby enhancing resilience against stressors [182]. They also activate plants’ defense mechanisms more effectively, enhancing their response to pest attacks or pathogen infections [183]. Bio-based products have been effective in mitigating abiotic stresses like drought, salinity, and heavy metal toxicity, such as brassinosteroids, which improve salt tolerance in crops. They also function as biocontrol agents against pathogens and pests, such as *Trichoderma* and Bacillus species, which enhance plant health while suppressing harmful organisms, reducing the need for synthetic pesticides [184]. Integrating bio-based products into agricultural practices supports sustainable farming by reducing reliance on chemical fertilizers and pesticides, promoting crop health and environmental sustainability by minimizing chemical runoff and soil degradation [185]. Overall, bio-based products offer a multifaceted approach to improving plant stress tolerance, contributing to enhanced agricultural productivity and sustainability.

## 14. Success Story of Gene Editing Tools for Biotic/Abiotic Stresses in Plants

Traditional techniques like crossbreeding and mutation breeding introduce genetic variability to select stress-tolerant phenotypes but are time-consuming and dependent on natural genetic diversity [186]. Transgenic methods, which involve inserting foreign genes for stress resistance, face regulatory hurdles and public resistance [187]. However, gene editing in plants for resistance to abiotic and biotic stresses involves the modification of genetic material to enhance resilience against factors such as drought, salinity, pathogens, and pests. Gene editing tools like CRISPR/Cas9 enable precise genetic alterations to improve stress tolerance, crop yield, and adaptability, addressing the challenges posed by environmental changes and agricultural demands [188]. Recent advancements such as CRISPR/Cas systems (e.g., CRISPR-Cas9 and CRISPR-Cas12) offer precise and efficient gene editing for enhancing resilience to biotic and abiotic stresses like salinity and pathogens [189]. Omics-based strategies, integrating genomics, transcriptomics, and metabolomics with AI-driven insights, facilitate the identification of stress-resilient genes for targeted editing [190]. TALENs (transcription activator-like effector nucleases) and ZFNs (zinc finger nucleases) are also powerful tools for gene editing in plants, helping them cope with challenges like pests, diseases, drought, and soil salinity. TALENs work by using specially designed proteins to target specific DNA sequences, while ZFNs use zinc finger proteins for the same purpose. Both create precise cuts in the DNA to either disable harmful genes or introduce beneficial ones, enabling scientists to develop crops that are more resilient to stress and better suited for growing in tough conditions [191] (Table 2).

### 14.1. CRISPR-CAS9-Based Gene Editing for Stress-Tolerant Plants

Gene editing technology has emerged as a powerful tool for enhancing plant resistance to both biotic and abiotic stresses. One notable example is the tomato plant, which is highly susceptible to viral, fungal, and bacterial infections. By modifying antiviral genes using CRISPR/Cas9 technology, researchers have successfully engineered tomato plants resistant to tomato yellow leaf curl virus (TYLCV) [197]. For fungal resistance, targeted gene modifications, such as *DMR6* and *Mlo1*, have been employed to develop resistant mutants without compromising plant growth or productivity [198]. In response to abiotic stress, gene editing has also improved heat tolerance. For instance, modification of the SLAGAMOUS-LIKE6 (SIAGL6) gene via CRISPR/Cas9 has enhanced fruit setting under high-temperature conditions, ensuring yield stability in tomato plants [67].

**Figure 3:**
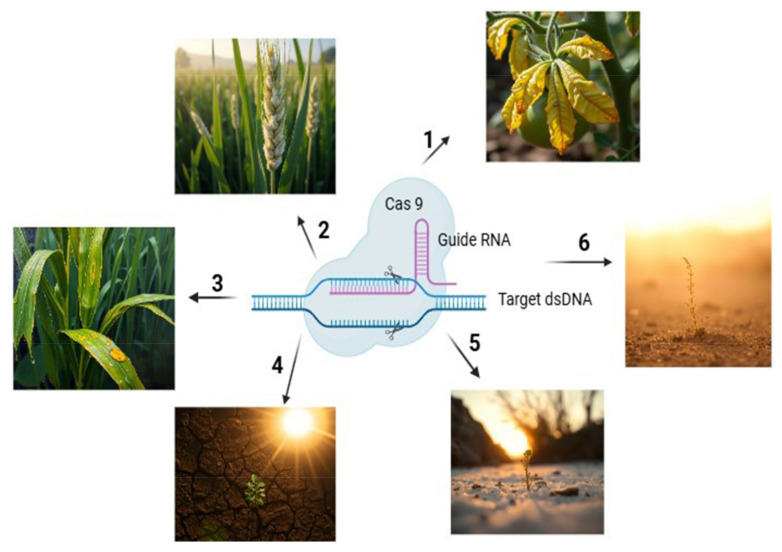
Use of CRISPR/CAS9 in crop improvement against abiotic/biotic stresses. (1) Tomato leaf curl disease, (2) powdery mildew infection in wheat plant, (3) rice plant infested with *Xanthomonas oryzae,* (4) drought, (5) high salinity, and (6) high temperature.

Crop improvement can be achieved by enhancing resistance to biotic and abiotic stresses (Figure 3). For instance, *Fusarium* wilt-resistant tomato plants were developed by targeting the *Solyc08g075770* gene, while powdery mildew resistance in tomatoes, such as the Tomelo variety, was achieved by editing the *SlMlo1* gene [199,200]. In rice, editing SWEET genes reduced susceptibility to bacterial infections caused by *Xanthomonas oryzae pv. Oryzae* [201]. Similarly, resistance to bacterial pathogens such as *P. syringae* has been achieved by modifying genes like *JAZ2* [202]. These breakthroughs underscore the potential of CRISPR/Cas9 in protecting crops from biotic stress. Moreover, knocking out of the OsNramp5 gene in rice significantly reduced cadmium accumulation as well, as it has been applied to minimize arsenic and cadmium uptake in other crops [203]. The use of multiplex editing further enables simultaneous targeting of multiple genes like BZR1, which regulates brassinosteroid-mediated stress responses and aids in high-temperature tolerance in tomatoes [204]. Similarly, CBF1 protects against cold stress, with CRISPR-induced cbf1 mutants showing increased resistance against chilling damage [205].

### 14.2. ZFN- and TALEN-Based Gene Editing for Stress-Tolerant Plants

**Figure 4:**
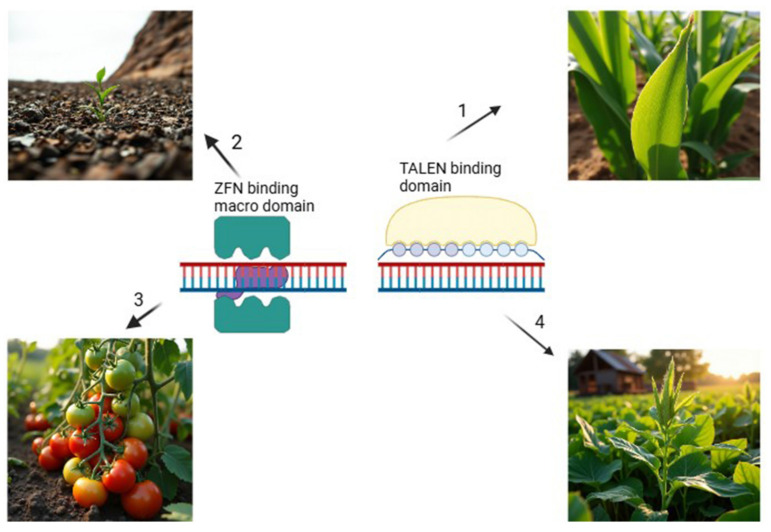
Applications of ZFN and TALEN on plants against abiotic/biotic stresses: (1) resistance against viruses in maize, (2) resistance of plants to heavy metals, (3) high-yield crops, and (4) herbicide resistance plant.

Through electroporation-based transient transformation-targeting genes like L1L4, ZFNs achieved high mutagenesis efficiency, leading to changes in leaf, flower, and fruit characteristics in tomato research for precise DNA editing, enhancing traits like flavor, nutrition, shelf life, pest resistance, and yield (Figure 4) [206]. Moreover, plants like tobacco, maize, and *Arabidopsis* are improved by inducing mutations that result in the creation of herbicide-resistant strains. They have also been applied for virus interference by targeting specific DNA sequences. Even though editing of the genome has been performed using ZFNs, challenges in cloning and designing ZFNs and their cost have led to the exploration of other genome editing tools [207]. Transcription activator-like effector nucleases (TALENs) were previously used to edit the *TaMLO* gene in wheat, conferring resistance to powdery mildew [208]. In recent times, CRISPR/CAS9 replaced ZFN and TALEN due to their simplicity, efficiency, and cost-effectiveness.

## 15. Future Prospects

Research on genome editing has the potential to revolutionize agricultural practices through its wide-ranging applications. One of the most notable benefits is the development of crop varieties that are more resilient to abiotic stresses like drought, salinity, and extreme temperatures. This increased resilience is essential for maintaining food production as climate change exacerbates these challenges. By enhancing the stress tolerance of crops, genome editing can also contribute to greater food security, especially in regions vulnerable to climate-related threats, ensuring stable food supplies for a growing global population. In addition to resilience, genome editing supports sustainable agricultural practices by enabling the creation of crops that are naturally more resistant to pests and diseases. This reduces the reliance on chemical inputs such as fertilizers and pesticides, leading to lower environmental impacts and more eco-friendly farming methods. The precision and efficiency of genome editing further allow for faster breeding cycles, enabling farmers to adapt quickly to changing environmental conditions, a process that traditional breeding methods cannot match. From an economic perspective, technology offers substantial benefits by improving yields and producing quality, which can enhance farmers’ profitability. Reducing crop losses due to abiotic stresses also ensures more stable incomes for agricultural communities. Moreover, genome editing is likely to face fewer regulatory hurdles compared to traditional genetic modification techniques, as its outcomes often resemble natural mutations. This aspect may lead to quicker adoption and increased acceptance among consumers and farmers alike. The integration of genome editing with other technologies, such as precision agriculture and marker-assisted selection, can further amplify its impact, creating holistic strategies to improve crop performance and sustainability. Overall, the advancements in genome editing research hold immense promises for transforming agriculture, ensuring food security, and addressing the pressing challenges posed by a changing climate [209].

Moreover, emphasis is placed on utilizing CRISPR/Cas technology in plant science, emphasizing its role in enhancing crop resilience and addressing critical agricultural challenges. One significant avenue is the application of this technology to a wider variety of crops, including under-utilized and minor species, as their genomes become sequenced [210]. This expansion could lead to the creation of resilient crop varieties that address diverse agricultural needs. Additionally, the ability to perform multiplex genome editing, where multiple genes are targeted simultaneously, offers opportunities to improve complex traits and streamline breeding programs for developing crops with multiple beneficial characteristics. Advancing the understanding of plant–pathogen interactions is another critical focus. Deeper insights into these molecular mechanisms will enable targeted genetic modifications to strengthen plant defenses against pathogens. Addressing regulatory hurdles and public concerns about genetically modified organisms (GMOs) is equally important [210]. Transparent communication with stakeholders and the public will be essential to build trust and ensure the acceptance of CRISPR-edited crops. Efforts to improve the precision of CRISPR/Cas technology and reduce off-target effects are ongoing. Developing refined tools and methodologies will enhance the reliability and safety of this approach in agricultural applications. Moreover, integrating CRISPR/Cas with other technologies, such as gene stacking and traditional breeding methods, could result in the development of robust crop varieties capable of enduring diverse environmental challenges. As climate change increasingly impacts agriculture, there is a pressing need to develop crops that can withstand extreme weather conditions, including drought, flooding, and temperature variability. CRISPR/Cas technology holds immense potential for creating climate-resilient crop varieties, offering a sustainable solution to these global challenges. These future directions underscore the transformative impact of CRISPR/Cas on agriculture, fostering food security and supporting sustainable farming practices.

## Figures and Tables

**Table 1:** Role of transcriptional networks against plant abiotic/biotic stresses.

Name of the Gene	Tolerance	Roles in Stress Response	Omics Approaches	References
*OsDREB1B*	Cold	Cold stress-induced upregulation of TF expression	Transcriptomics	[88,148]
*OsDREB1* TFs *OsDREB1A*, *OsDREB1C*	Cold, Drought, Salt	Proline accumulation and control of the expression of genes that respond to stress	Transcriptomics, proteomics	[88,148]
*OsDREB1G*	Drought, Cold	Attaching to the DRE component	Transcriptomics, ChIP-seq	[88,149]
*OsDREB2B*	Heat	Control of gene expression through alternative splicing brought on by stress	Transcriptomics, RNA-seq	[150]
*OsMYB3R-2*	Cold, Drought, Salt	Control of gene expression unique to the G2/M phase	Transcriptomics, co-expression analysis	[151]
*SNAC1*	Drought, Cold, Salt	An increase in the expression of genes related to stress tolerance and stomatal closure	Transcriptomics, functional genomics	[152,153]
*OsDREB1F*	Drought, Salt, Cold	Control of the expression of ABA-responsive genes	Transcriptomics, ChIP-seq	[154]
*OsNAC10*	Drought	Increasing grain production under drought	Transcriptomics, functional genomics	[155]
*AP37*	Drought	Increased grain yield	Transcriptomics, gene expression analysis	[152]
*ZFP252*	Drought, Salt	Buildup of sugars and proline	Transcriptomics, metabolomics	[156]
*AP59*	Drought	Spikelet disruption	Transcriptomics (RNA-seq)	[152]
*ZFP245*	Drought, Cold	Increased activity of the ROS enzyme	Transcriptomics, metabolomics	[157]
*OsMYB4*	Chilling	The TF decreases membrane injury	Transcriptomics (RNA-seq), functional genomics	[158]
*DST*	Salt	H_2_O_2_-induced regulation of stomatal closure	Transcriptomics, functional genomics (mutant analysis)	[159]
*OsNAC6*/*SNAC2*	Drought, Salt	Control of the expression of genes responsive to biotic and abiotic stress	Transcriptomics, ChIP-seq	[4,152,153]

**Table 2:** Plant genome/gene editing tools against abiotic/biotic stressors.

Gene Editing Tool	Example	Gene Targeting and Modification for Stress Resistance	Regulation of Stress-Responsive Proteins and Pathways	References
CRISPR/Cas9	Improving tomato traits	Targeted gene editing to enhance resistance to heat, cold, drought and salinity by modifying stress-related genes.	Modifies stress-responsive proteins involved in heat, cold, drought, and salinity resistance.	[192]
CRISPR/Cas9	Hybrid proline-rich proteins in crops	Disturbing negative regulators at the genetic level for multi-stress tolerance.	Alters proline-rich proteins, disrupting stress-regulating protein pathways for enhanced tolerance.	[193]
CRISPR/Cas9	Various crop improvements	Enables precise gene targeting with minimal off-target effects, simplifying genome editing.	Affects protein expression related to plant growth, stress response, and metabolism.	[6]
TALENs, CRISPR/Cas9	SWEET sugar transporters	Engineering SWEET genes to regulate abiotic stress responses such as drought and salinity.	Regulates sugar transporter protein to improve stress response at the proteomic level.	[194]
CRISPR/Cas	Multiple abiotic and biotic stresses	Edits genes involved in fungal, viral, and bacterial resistance and abiotic factors like herbicides and drought.	Modifies plant immune proteins and enzymes that combat fungal, viral, and bacterial infection.	[186]
CRISPR/Cas	Abiotic stress tolerance in rice and Arabidopsis	Modifies genes regulating tolerance to salinity, temperature extremes, and other abiotic factors.	Regulates stress-associated proteins like ion transporters and heat shock protein.	[195]
Genetic Modification	Climate-smart banana development	Alters genes to improve resistance to abiotic and biotic stress.	Enhances protein networks linked to resistance against environmental and pathogen-induced stress.	[196]
